# A Comprehensive Dataset for Activity of Daily Living (ADL) Research Compiled by Unifying and Processing Multiple Data Sources

**DOI:** 10.3390/jpm15050210

**Published:** 2025-05-21

**Authors:** Jaime Pabón, Daniel Gómez, Jesús D. Cerón, Ricardo Salazar-Cabrera, Diego M. López, Bernd Blobel

**Affiliations:** 1 Telematics Engineering Research Group, Telematics Department, Universidad del Cauca, Popayán 190002, Colombia; japabon216@unicauca.edu.co (J.P.); dgomez216@unicauca.edu.co (D.G.); jesusceron@unicauca.edu.co (J.D.C.); ricardosalazarc@unicauca.edu.co (R.S.-C.); dmlopez@unicauca.ed.co (D.M.L.); 2 Medical Faculty, University of Regensburg, 93053 Regensburg, Germany; 3 eHealth Competence Center Bavaria, Deggendorf Institute of Technology, 94469 Deggendorf, Germany; 4 First Medical Faculty, Charles University Prague, 12800 Prague, Czech Republic

**Keywords:** activity of daily living, human activity recognition, data preparation, dataset integration, machine learning

## Abstract

**Background**: Activities of Daily Living (ADLs) are essential tasks performed at home and used in healthcare to monitor sedentary behavior, track rehabilitation therapy, and monitor chronic obstructive pulmonary disease. The Barthel Index, used by healthcare professionals, has limitations due to its subjectivity. Human activity recognition (HAR) is a more accurate method using Information and Communication Technologies (ICTs) to assess ADLs more accurately. This work aims to create a singular, adaptable, and heterogeneous ADL dataset that integrates information from various sources, ensuring a rich representation of different individuals and environments. **Methods**: A literature review was conducted in Scopus, the University of California Irvine (UCI) Machine Learning Repository, Google Dataset Search, and the University of Cauca Repository to obtain datasets related to ADLs. Inclusion criteria were defined, and a list of dataset characteristics was made to integrate multiple datasets. Twenty-nine datasets were identified, including data from various accelerometers, gyroscopes, inclinometers, and heart rate monitors. These datasets were classified and analyzed from the review. Tasks such as dataset selection, categorization, analysis, cleaning, normalization, and data integration were performed. **Results:** The resulting unified dataset contained 238,990 samples, 56 activities, and 52 columns. The integrated dataset features a wealth of information from diverse individuals and environments, improving its adaptability for various applications. **Conclusions:** In particular, it can be used in various data science projects related to ADL and HAR, and due to the integration of diverse data sources, it is potentially useful in addressing bias in and improving the generalizability of machine learning models.

## 1. Introduction

This paper extends the consideration of aspects published in earlier *pHealth* Proceedings [1,2,3]. Activities of Daily Living (ADLs) encompass essential and routine tasks performed at home, such as walking, dressing, and feeding without assistance [4]. The growing use of ADL concepts in healthcare, driven by technological advancements, extends beyond assessing older adults’ independence to areas like sedentary behavior monitoring, rehabilitation therapy tracking, delirium risk identification, and chronic obstructive pulmonary disease monitoring [5,6,7,8].

The relationship between ADL identification and patient lifetime health records is relevant for improving patient care outcomes. ADL identification is crucial, in this context, for assessing a patient’s functional capacity and personalizing their care, especially in patients with chronic illnesses or who may be experiencing functional decline. This identification helps determine whether a patient needs assistance with basic tasks such as dressing, eating, or bathing [9,10,11,12,13].

Although healthcare professionals often rely on the Barthel Index for assessments of ADLs [14], its limitations, including reliance on patient memory and potential misinformation, underscore the need for more reliable methods. This gap is addressed by leveraging Information and Communication Technologies (ICT), specifically human activity recognition (HAR).

Human activity recognition (HAR) employs sensors such as cameras, accelerometers, and gyroscopes to collect and analyze relevant datasets [15,16,17,18,19,20]. This article explores datasets derived from HAR research, integrating accelerometers, gyroscopes, inclinometers, and heart rate monitors. These datasets play a crucial role in developing machine learning (ML) models to predict, monitor, and support people in aspects related to ADL [21].

Although HAR could detect simple or primitive activities, such as walking, sitting, or standing, and the Barthel Index is related to more complex activities, such as grooming, dressing, toileting, mobility, continence, and feeding, it is possible, through a context analysis (the location of the person in the house, the time of day, and/or their most recent trip) and the simple activity identified with HAR, to infer, through an ML model, what complex activity the person is performing (for example dressing or feeding).

The research aims to merge identified datasets into a coherent and complete ADL dataset, facilitating future research in machine learning applied to human activity recognition (HAR). This goal involves extensive dataset searching, meticulous data preparation, and integration of multiple datasets into a unified ADL dataset. The article presents the procedure performed to obtain the unified ADL dataset. This procedure and the generated dataset are considered the most relevant products of the work.

The procedure above was based on the data science methodology named CRISP-DM since it has been the methodology most commonly applied in this type of project since 2007 [22]. CRISP-DM proposes the development of six phases—business understanding, data understanding, data preparation, modeling, evaluation, and deployment. The above procedure for obtaining an integrated dataset in ADL focuses on stages two and three (named data understanding and data preparation). Stages 1, 4, 5, and 6 of CRISP-DM can be performed depending on the specific goal of the ADL-based data project to be implemented. Using the proposed procedure, the generated dataset will be used to perform various data science projects related to ADL and HAR.

In the data understanding stage, CRISP-DM proposes certain tasks, among which the selection and categorization of the dataset stand out. These tasks are presented in Section 2 and Section 3 of the document. In the data preparation stage, CRISP-DM proposes tasks such as the following: the analysis of dataset characteristics; cleaning and preparing the datasets; dataset normalization; and the structuring, integration, and formatting of the data. These tasks are presented in Section 4 of the paper. The characteristics of the unified dataset are presented in Section 5 of the document. Finally, the conclusions of the article are presented in Section 7.

## 2. Dataset Selection

The dataset selection method considered relevance, representativeness, quality, and accessibility. Datasets not older than 12 years were considered relevant due to the consolidation of ADL research during this timeframe. Representativity, quality, and accessibility were guaranteed by the search of the literature and the main dataset repositories, which are internationally recognized and have adequate accessibility. The dataset selection method followed is detailed below.

The search was performed considering documents published since 2012 because the principal objective was obtaining as many datasets as possible related to ADLs and meeting the established inclusion criteria (Table 1). The datasets were searched for in the following databases: Scopus, UCI Machine Learning Repository, Google Dataset Search, and the University of Cauca Repository. The search strategy was built with the following keywords: “TITLE-ABS-KEY ((“open dataset”) AND ((“HAR”) OR (“ADL”)))”. In particular, to search in the UCI Machine Learning Repository, the following search strings were used: “ADL”, “SHAR”, and “HAR”.

Certain inclusion criteria were defined in the search for datasets, which were key to integrating a single dataset. A list of dataset characteristics was initially made to obtain the inclusion criteria presented in Table 1.

The list of characteristics identified three inclusion criteria for the datasets, which are presented in Table 2. The definition of the criteria and their application ensured a consistent and efficient selection of datasets for this study.

The feature analysis technique of the DESMET method was chosen as the search and selection technique for the datasets. DESMET is a multi-component method comprising guidelines for conducting various evaluation activities, such as selecting evaluation methods, quantitative case studies, quantitative experiments, and characteristic analysis [23]. Feature analysis is a flexible technique for evaluating software engineering methods and tools. It involves identifying a list of required or desirable product features and assigning a score to each product based on those features. This technique facilitated the creation of the inclusion criteria and the choice of datasets for this project.

Table 3 presents the datasets identified through each platform that met the dataset research criteria. A total of 29 datasets were identified.

Considering the defined requirements (Table 1), the evaluation process resulted in four distinct cases representing the quantitative aspect of the search:

- Case 1. This scenario represents the ideal case where all requirements were fully satisfied, resulting in a score of 3. Such datasets were immediately selected as they met all the necessary criteria.
- Case 2. Two of the three requirements were fulfilled in this case, leading to a score of 2. This situation introduces a quantitative approach alongside qualitative analysis. The dataset’s information and potential benefits were carefully considered to determine suitability.
- Case 3. Only one of the three requirements was met, resulting in a score of 1. Due to the lack of comprehensive compliance, datasets in this category were promptly discarded.
- Case 4. Datasets failing to meet any of the requirements receive a score of 0.

Datasets with a score of 3 were selected without any qualitative analysis; datasets with a score of 2 were subjected to further analysis (a detailed qualitative analysis). Finally, datasets with scores of 1 or 0 were excluded without further consideration. It is noteworthy that there were no datasets with a rating of 0. Table 4 summarizes the results of the qualification of the datasets found.

Finally, after performing the qualitative analysis of the 3 datasets with a score of 2, it was decided to discard them as well. Therefore, a set of 20 datasets, with their categorization task performed, was obtained and is presented in the following section.

## 3. Dataset Categorization

Once the 20 datasets were selected, a characterization was performed with the information based on the identifier number, the name of the dataset, its description, activities (activity number, activity name, and activity duration), population (average age, average height, average weight, restrictions, total people, and notes), devices (device number, device name, device brand, device location, device sensor, units, sensor brand, sampling rate, controlled, uncontrolled, and activity location), dataset weight, and additional notes. Table 5 presents a summary of the characterization of the datasets, highlighting the main features.

## 4. Data Preparation

In this phase, various techniques were used to analyze, transform, clean, and structure the data to ensure it is useful and relevant to the problem. The aforementioned problem relates to merging the identified datasets into a coherent and comprehensive ADL dataset, facilitating future research in machine learning applied to HAR. Several aspects make the selected datasets not sufficiently similar for simple integration; all of these aspects were considered, and certain tasks were established to make the necessary adjustments. The tasks performed in this preparation phase are detailed below.

### 4.1. Analysis of Dataset Features

Feature analysis is essential in data science because it allows one to discover patterns, test hypotheses, and make data-informed decisions. Due to the number of analyzed datasets, several quantitative features were chosen to guarantee correct data integration. The following variables were chosen for data harmonization: sensors, units, frequency, activities, and sensor location.

#### 4.1.1. Activities

**Figure 1:**
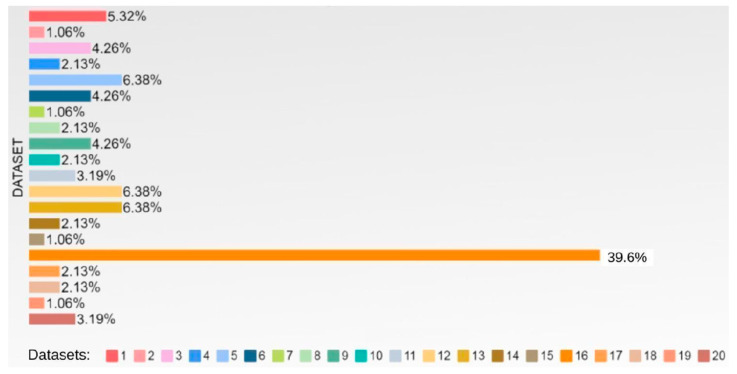
Number of activities per dataset.

One of the most crucial aspects of this project is the classification of activities. The 20 datasets contained 293 activities. Dataset 16 stands out because it has 116 activities, which corresponds to 39.6% of the total activities identified in all the datasets. Figure 1 shows a percentage distribution of the activities in the datasets.

The most frequent activities in the datasets were analyzed. Table 6 shows the number of common activities in each dataset. Six activities are the most repeated in the datasets, between 7 and 14 times. The “walk” activity is the most frequent in the 14 datasets.

#### 4.1.2. Sensors

Similarly to what was carried out for the activities in the 20 datasets, the sensors used in each of the 20 previous works were reviewed to determine the sensors used in each case. The main sensors used were the accelerometer, used in all 20 cases, and the gyroscope, used in 19 out of the 20 cases. Another sensor used in many cases was the magnetometer, used in 7 of the 20 cases. In each case, the other sensors had fewer repetitions, between 1 and 2 times.

#### 4.1.3. Units of Measurement

In the measurements used by the sensors (mainly the accelerometers and gyroscopes), different units were used in the 20 datasets; for this reason, a normalization of these units was required. The performed normalization is detailed in Section 4.3.

#### 4.1.4. Frequency

To facilitate data processing, it was necessary to harmonize the frequencies at which the data had been taken in each dataset. The mode frequency of all 20 datasets was 50 Hz, so it was decided to use this value for all datasets, applying a resampling technique that is explained in detail in Section 4.2.2.

#### 4.1.5. Sensor Location

The sensors used to collect the data in each of the 20 datasets were located on different parts of the body of the person who performed the ADL. An analysis was developed to identify the parts of the body most used for the location of the sensors. In 10 of the 20 cases, at least one of the sensors was located on the right wrist. In 4 of the 20 cases, at least one of the sensors was located on one of the shoes. There were other locations with 3 cases each in the 20 datasets: hip, chest, waist, and right pocket. The remaining locations varied across the 20 cases reviewed (appearing only once or twice).

#### 4.1.6. Defining Unified Dataset Columns

Considering the performed analysis of dataset features (in Section 4.1.5), the features of the unified dataset were defined. In the data cleaning process, each dataset was adjusted to comply with the structure shown in Table 7. The following aspects were considered when selecting these features:

- Identifying the dataset from which the record was obtained in the final dataset was initially considered necessary.
- The activity identifier was necessary in order to identify the activity associated with the respective record.
- The final dataset contained key features, including data on the X, Y, and Z axes of the gyroscope and accelerometer sensors.
- The “code” feature was initially considered important, as it integrated the “sensor type”, “location side”, and “sensor location” data. These data are essential for understanding how the record data were measured.
- Timestamp data was a relevant feature for each record in the various datasets.
- Frequency was also a relevant feature, as it is very important to determine the frequency with which the records were collected in the initial datasets.

**Table 7:** Unified dataset features: activity logs and sensor measurements.

Columns	Description	Data Type
Dataset number	Dataset numeric identifier	Integer
Activity	Activity identifier	Integer/Subject
X_Acc	X-axis acceleration measurement	Float
Y_Acc	Y-axis acceleration measurement	Float
Z_Acc	Z-axis acceleration measurement	Float
X_Gyro	X-axis gyroscope measurement	Float
Y_ Gyro	Y-axis gyroscope measurement	Float
Z_ Gyro	Z-axis gyroscope measurement	Float
Code	3 identification digits for sensor features	Int
TimeStamp	Sample timestamp	Date/Time
Frequency	The frequency at which data are taken	Float
User	A person who performed the activity	Integer
Trial	Activity test number	Integer

In the feature analysis, the need to have a column called “Code” was considered. This column consists of 3 identification digits. This code facilitated the dataset integration analysis because it comprises sensor type, location side, and sensor location. Table 8 presents how the coding is configured. An example of a 3-digit code would be 113; this code means that the sensor type is a gyroscope (for the first digit equal to 1), the location side is right (for the second digit equal to 1), and that the sensor location is foot (for the third digit equal to 3).

### 4.2. Cleaning, Preparation, and Integration of Datasets

This data science task is usually one of the most laborious due to its complexity. In this work, each dataset had its own information distribution, which implied specific cleaning and standardization procedures.

#### 4.2.1. Cleaning of Datasets

Some datasets were delivered as multiple .csv files in a single folder. In contrast, others were scattered across different folders and subfolders and with various file extensions (such as .txt, .mat, .csv, .aff, .dat, etc.) without clear raw data documentation. Unfortunately, for data quality reasons, some of the 20 original datasets had to be discarded, ultimately leaving 15 datasets for the analysis.

For each of the datasets, the following three steps were implemented.

- Find a paper that describes how the dataset was constructed. The description must include complete information about sensor specifications and the type of measurement units to provide a clearer context for the chosen dataset. Not all datasets had a paper, and none provided all the required information.
- If the dataset did not have a reference paper or provided enough information, the “readme” file that some datasets had or the descriptions of the data attached within a file with the sensor measurements were reviewed. Other papers and/or websites where information on the dataset could potentially be found were also searched. At times, the information found was insufficient or was claimed to be on discontinued websites. In these cases, an attempt was made to contact the authors directly.
- Considering the completion of steps 1 and 2 and the context of the datasets, some datasets were discarded. The discarded datasets are specified later.

For each of the datasets, the downloading of a large number of files in different formats was required. The “code” column explained in Section 4.1.6 (Table 8) was complex to obtain for each dataset since the information was obtained from different fields (sensor type, location side, and sensor location were found in various fields of the datasets). For some of the datasets, it was necessary to perform some algorithms to facilitate downloading many files in different folders and unifying them into a single one. Algorithms were useful to automate this task (downloading and organizing the files) because completing it manually would have taken too much time and resources.

Below, the five discarded datasets are detailed, with their respective justifications, finally providing 15 of the initial 20 datasets.

The “IMU dataset: Walking activity recognition using inertial measurement unit modules” ([30]) was discarded due to its inherent ambiguity. The “Human activity recognition using smartphones dataset” ([33]) was discarded because we identified that it is a previous version of the dataset (number 10) that has already been included [32]. The “Smartphone dataset for human activity recognition (HAR) in ambient assisted living (AAL) dataset” ([37]) was discarded because we found incomplete data. The available information focused on the processed data without specifying the number of samples used in feature extraction. The labels were only associated with the processed samples. In the documentation, there was no information about how many raw data samples were taken for feature extraction. The datasets “Simultaneous indoor pedestrian localization and house mapping based on inertial measurement unit and Bluetooth low-energy beacon data” [41] and “Indoor trajectory reconstruction of walking, jogging, and running activities based on a foot-mounted inertial pedestrian dead-reckoning system” ([42]) were discarded because, through a detailed review of the data, it was identified that they focused on the reconstruction of users’ trajectories, which has no direct relationship with ADL.

#### 4.2.2. The Preparation of the Datasets

As discussed in Section 4.1.4, resampling was necessary to address the issue of frequency disparity. Frequency disparity refers to situations where there is a significant disparity in the number of observations or events recorded across different time intervals [44]. In the datasets under consideration, data were collected at varying frequencies, leading to an uneven distribution of observations over time [45]. This can skew analysis and model performance, as certain periods may be overrepresented while others are underrepresented. The resampling method is presented as a fundamental solution. A key advantage was achieved by standardizing the sampling frequency of all datasets, thereby eliminating concerns related to temporal discrepancies. This significantly simplifies the segmentation process and improves the overall consistency of the selected data.

Temporal resampling modifies the temporal frequency of data by adding or deleting records in a minority or majority class, respectively [46]. Two resampling techniques, oversampling and undersampling, were implemented [47]. Oversampling involves adding records to a minority class, and undersampling removes records from the majority class.

The most common frequency in the datasets is 50 Hz. After the cleaning process and the necessary subdivisions of the datasets, the frequency mode continues to be 50 Hz. Therefore, 50 Hz was determined as the target frequency for all datasets. To homogenize the frequency at 50 Hz in the selected datasets, oversampling was implemented in those with a frequency lower than 50 Hz and undersampling in the sets with a frequency higher than 50 Hz.

To verify the resulting data after applying resampling and to avoid bias, it was evaluated that the data maintained its distribution: median, standard deviation, and average. We used the Pandas Data Frame function/method called “describe”, which provided a concise summary of the statistics, which includes the count of non-null values, the mean that shows the average value, the standard deviation that measures the dispersion of the data, the minimum and maximum value in each numerical column, and the quartiles that indicate how the data are distributed.

Regarding the normalization of dataset units, ensuring the consistency of units in a dataset that includes multiple sources is essential. For effective comparison and analysis, all data must be in the same unit of measurement. This not only benefits uniformity in data scale but also enhances precision in analysis. In machine learning algorithms and other data analysis techniques, the general assumption is that data are presented in uniform units. Unit variations could introduce uncertainty into the data, which could lead to misclassifications by the models. Due to the above, it was decided that the units of the datasets would be presented in m/s^2^ and degrees/second (or dps).

Four out of the 15 selected datasets encountered issues with their measurement units. Specifically, datasets 11, 12, and 16 from Table 6 lacked specifications for some of their measurements, requiring communication with the authors for clarification. In certain cases, assumptions were made regarding the unit used, and data congruency was verified. Moreover, dataset 15 from Table 6 indicated a certain unit for one of its measurements, but the data did not match up. The authors were contacted, and the correct unit information was provided. Despite the significant time investment, the unification of all measurement units across the datasets was ultimately achieved.

Another task in the data preparation phase was cleaning outlier data. All datasets had relatively low counts of outliers, so their removal was simple. Functions were used to identify these values, then they were eliminated, and the calculation of new, more representative minimum and maximum values was verified. In datasets 11 and 16 (Table 6), records were found without information in the target field (or label). Therefore, they were also eliminated.

Due to the repeated activities in different datasets, it was necessary to differentiate the activities by Dataset ID and Activity ID; for example, in all datasets, the activity “Walking” now had the identifier number 6. Also, for activities that did not have clear information in their description (such as the activity called “Active” in dataset 2, which meant that the person was moving without specifying the type of activity) or activities that were not identified as ADL, instead of an ID, a letter “X” was placed for analysis and finally its possible elimination. Initially, there were 308 activities; once the mentioned activities were eliminated, 92 remained. This number of activities was initially considered adequate for the unified dataset.

#### 4.2.3. Integration of Datasets

A vertical concatenation was performed to unite all the datasets, thus obtaining 263,884,250 samples and 13 columns weighing 23.54 GB. Unfortunately, because it is a large dataset, it was not possible to process it, and it was not even possible to load it into memory using the Pandas pd.read_csv() library. For this reason, it was necessary to process dataset 16 (in Table 6), as it has the highest percentage of total records, to reduce the number of records. Reducing the number of records implied a reduction in the number of users and, further, a reduction in the number of activities in the dataset. Moreover, a more robust analysis (for example, activities such as “jogging”, “running”, “light skipping”, and “jumping rope” were combined into a single category), which contributed to reducing the number of activities in the dataset, as did reducing the number of locations in the sensors of the dataset.

Finally, a unified dataset of 127,766,944 samples, 56 activities, and 11.88 GB in size was obtained. This size allowed for the required processing.

### 4.3. The Normalization of the Integrated Dataset

Considering that the ranges of the accelerometer and gyroscope measurements in the different datasets were unequal, a normalization process was performed. In so doing, data biases were also avoided, because more representative data could be obtained, simulating data coming from a single source or a uniform dataset. The selected normalization type is known as Z-score [48]. This technique transforms the data so that the average is zero and the standard deviation is one. To achieve this, the following equation is used:

*Z* = (*x* − *μ*)/*σ* (1)

where

*Z*: the punctuation.

*x*: the variable to transform.

*μ*: the average value of all samples of the variable to be transformed.

*σ*: the standard deviation of all samples of the variable to be transformed.

After the normalization process was completed, the size of the dataset was 14.04 GB.

### 4.4. Structuring, Integration, and Formatting of Data

The articles consulted in the literature review showed the convenience of treating the data with feature extraction instead of the raw data. This technique (feature extraction) reduces redundant data, increases the learning speed, and more efficiently uses computational resources. Additionally, in many cases, the accuracy of the models improves.

This technique consists of training the models with raw data features based on measurements such as the mean, median, and standard deviation, among others. The process for data structuration has 2 stages—dataset segmentation and feature extraction. Feature extraction includes feature selection, feature extraction, application, and binding. The stages mentioned are presented in detail below.

#### 4.4.1. Dataset Segmentation

Features are typically calculated for segments of a dataset. These segments must be uniform and coherent to have the same features. The authors of the work determined that the segments are 10 s, which is the time people could finish performing a certain activity without any inconvenience. Below are three aspects that were taken into account in the segmentation process:

- The dataset has a frequency of 50 Hz. The segmentation was performed with 500 samples (corresponding to 10 s of data). However, it was evident that, due to the nature of the organization of the activities of the unified dataset, it is borne in mind that some segments could describe more than one activity.
- To solve this issue of activities in more than 1 segment, the unified dataset was ordered by activity (instead of by each dataset that made it up). After the samples of each activity that prevented it from being a multiple of 500 were eliminated, this change ensured that when fragmenting the segments, each segment only refers to a certain activity—however, some segments combined data from more than one dataset.
- A code was developed to identify the segments with combined data (from more than one dataset). The code identified 16,516 segments, which were subsequently eliminated. One of the negative consequences of the elimination of the 16,516 segments was the loss of 2 datasets (datasets 2 and 15). We analyzed the significance of the absence of these 2 datasets, and the following conclusion was made: Because users labeled datasets 2 and 15, their values were low in reliability. Due to the above and considering that the loss represented only approximately 1% of the total data, this was finally considered manageable.

As a result, the final number of segments remained at 238,990, encompassing over 119 million samples.

#### 4.4.2. Feature Extraction

Feature extraction is divided into two parts—feature selection, which explains how the features were selected, and the application and joining of feature extraction, which details how these features were applied to the segments of the datasets.

##### Feature Selection

We chose certain features that make describing and distinguishing between different activities easier. It is important to select these features carefully to ensure the training process goes smoothly. We ensured that there was no overlap between the features, so each one provided unique and useful information for each activity. The specific features that we selected are:

- Entropy;
- Average;
- Standard deviation;
- Maximum;
- Minimum;
- Mean;
- Absolute average;
- Resulting average—a general value is calculated in all directions (x, y, z);
- Magnitude—a general value is calculated in all directions (x, y, z).

##### Application and Joining of Feature Extraction

Using the defined features, we calculated the relevant data for each segment, creating separate data frames for each feature, which were then joined horizontally. To enhance the training and understanding of the models, we separated the components of the “Code” column into three distinct columns: “Sensor_Type”, “Left_Right”, and “Location”. This increased the dataset’s size from 1 column to 3 columns, resulting in a final dataset with 238,990 samples, 52 columns, 56 activities, and a size of 135.7 MB. The size reduction led to significant benefits in terms of computational efficiency, training time, and model size.

## 5. Unified Dataset

As mentioned in the Introduction, the procedure performed to obtain the unified ADL dataset (described in Section 2, Section 3 and Section 4) and the generated dataset are considered the most relevant results of the work. The contents of the final dataset, obtained after performing the procedure mentioned in the previous sections, are described below.

The unified dataset comprises 238,990 samples, 56 activities, and 52 columns featuring segment number, dataset number, activity number, sensor_type, left_right, location, and 46 columns related to the selected features. It should be noted that the seven initial features (entropy, average, standard deviation, maximum, minimum, mean, and absolute average) were measured for each of the acceleration measurements (Acc) in every axis (x, y, z), as well as for each of the gyroscope measurements (Gyro) in each axis. Thus, there are 21 columns related to Acc and 21 to Gyro. The remaining two selected features (resulting average and magnitude) are general measurements in all directions, resulting in only one value for Acc and another for Gyro for each feature, adding another four columns to the 42 mentioned above, totaling 46 columns related to the selected features in the final dataset.

The final dataset, named the “multi-source, integrated and preprocessed ADL dataset” [49], was made available to the public via a link on the Kaggle platform. It is expected to be utilized in machine learning (ML) model generation processes to predict daily life activity.

## 6. Discussion

We obtained an ADL dataset that integrates information from various sources, ensuring a rich representation of different individuals and environments. The unified dataset, despite having different data collection methods, sensor types, and population demographics, is a comprehensive dataset with a broad number of records and activities, which can be used in various types of research related to ADL and HAR.

The datasets selected for the integration process and the creation of a single ADL dataset showed various activities. Although the sensors used were mostly the same (accelerometer and gyroscope), the data capture procedure varied significantly in some cases. This variety (mainly in terms of activities and capture procedures) required several adjustments and decisions in the integration process to find the best possible approximation to a unified and adaptable dataset. The procedure and the dataset finally generated are considered the main products of this work.

The data preparation phase, performed during the dataset integration process, was carried out by analyzing various options and searching for the best results, which is why a considerable period of time was required for its execution (more than 50% of the time allocated for the research). This preparation used various techniques to analyze, transform, clean, and structure the data. Analyzing the detailed information of some datasets, it was necessary to make decisions such as discarding some datasets (datasets 11 and 16) because some records did not have the target label, because they did not have a specification for some of their measurements, and because the number of activities in dataset 16 was too high, due to the high specification of the activities it handles. Additionally, it was necessary to perform tasks such as adjusting the data sampling frequency in some datasets and normalizing them. Section 4 of this document details all the steps to prepare the data from the different sources to ensure that the final dataset had adequate reliability and was as standardized as possible.

One aspect relevant to the results obtained is the potential bias that each data source and the generated dataset could have in terms of population (over-representing certain groups of individuals), environments (home vs. healthcare), or activities. However, using various data sources in a single dataset reduces the potential bias in most cases, since we are expanding the population, environments, and activities used by most datasets individually.

Additionally, regarding the aforementioned bias, we highlight that in the dataset categorization process (Section 3 of this document), several factors of each dataset were considered, such as activities (activity number, activity name, activity duration), population (average age, average height, average weight, restrictions, total people, notes), devices (device number, device brand, device location, device sensor, units, among others). We subsequently analyzed that a relevant aspect that was not considered was that the final dataset should include a wide and equitable range of real-world scenarios and environments. Although including a greater number of data sources broadens the range of these factors, this does not guarantee that the data are sufficiently balanced (equitable range) nor that there is an adequate number of real-world scenarios. Therefore, it is pertinent to consider this aspect as future work to reduce further the possible bias that may occur in the final dataset, in these factors, and to deal with the adequate balancing of the data.

Despite this analysis, the generated dataset may be useful in research projects that require testing the efficiency of a machine learning model responsible for predicting the ADL being performed at a given time. Given the considerably high number of activities and records, an appropriate training process could be implemented to improve the metrics of the evaluated model.

Regarding the limitations of this work, in addition to what has previously been mentioned regarding the possible bias in factors such as population, activities, and scenarios, and the imbalance of records concerning the type of activity, it is also important to highlight the following factors. First, regarding the databases used, although those selected are internationally recognized many other options could have been used, such as Zenodo. And second, regarding the computational resources available to the project, although they allowed for a robust and strict data preparation process, it is possible that with better features of these resources the results obtained could have been improved; mainly, regarding the number of possible records in the final dataset, and the duration of some steps in the preparation of the data.

Finally, we recommend to the researchers using the unified dataset that the type of variables, sensors, and activities selected should be carefully analyzed, which are detailed in the shared link of the dataset platform used [49]. Although we consider it a sufficiently broad range, some key factors are not included in the research. Three features that are important to consider in the final dataset are “sensor_type”, “left_right”, and “and location”, which refer to the type of sensor and the location on the body for data collection. Therefore, these features must be considered to obtain adequate results if an ML model is to be tested with the unified dataset generated in this research project.

## 7. Conclusions

Thanks to its richness and diversity, the final dataset presents immense potential for a diverse range of data science scenarios and projects. The significance of this diversity cannot be overstated, as it encompasses numerous users, activities, and locations. By selecting different activities or locations, versatile applications across various scenarios can be enabled.

Due to each dataset’s unique configuration and presentation, structuring, cleaning, and preparing it for the final dataset can be tedious and laborious. Understanding how each dataset was prepared individually is, therefore, essential for the scientific community.

Unifying disparate sources requires meticulous attention to detail. This article provides comprehensive documentation and explanation of the entire process, ensuring transparency and serving as a valuable resource for the scientific community. At the time of this research, a notable gap existed in the literature on preparing and fusing diverse datasets in ADL. This article aims to fill this void and provide valuable insights for addressing similar challenges in future research endeavors by detailing the methodology employed.

Regarding future work, it is important to highlight the importance of improving the inclusion of a wider range of real-world scenarios, environments, and activities in a possible future dataset, which seeks to integrate diverse data sources. Additionally, future dataset integration work could include more possible data sources and sufficient computational resources to handle a much larger volume of data. In future data integration processes, if there are problems with the size of the integrated dataset, it is suggested that the resulting dataset be processed in batches to avoid deleting records from a dataset or a complete dataset.

Finally, we propose developing some type of machine learning model or models that test their effectiveness in predicting ADL using the dataset generated in this research.

## Figures and Tables

**Table 1:** List of characteristics of datasets.

Characteristic Number	Dataset Characteristic
1	The datasets must be on the topic of ADL.
2	The activities of the datasets must be categorized and labeled so that they can be combined into a unified dataset and guarantee better algorithm performance.
3	The datasets must contain angular motion (gyroscope) and/or acceleration (accelerometer) data.
4	The size of the datasets was unrestricted because it was interesting to have a considerable amount of data to perform a substantial analysis of the algorithms.
5	The language and origin of the datasets are independent of the interest of the authors.
6	The format of the datasets is independent of the interest of the authors.

**Table 2:** Requirement criteria.

Requirement Number	Requirement Description
1	The dataset should strictly exclude any data originating from images or videos. Instead, it should emphasize sensor data, including those from accelerometers, gyroscopes, and magnetometers.
2	Activities within the dataset must be properly categorized and labeled, enabling the identification of specific actions such as walking, sitting, sleeping, and more.
3	The dataset must encompass a comprehensive range of activities associated with everyday life, covering a diverse set of actions performed regularly.

**Table 3:** Number of datasets found by each search platform.

Search Platform	Number of Datasets
Scopus	11
UCI	13
Google	2
University of Cauca	3

**Table 4:** Number of datasets per rating.

Score	Number of Datasets
3	20
2	3
1	6
0	0

**Table 5:** Summary of dataset characterization.

Dataset Number	Reference	First Author	Number of Detected Activities	Detected Activities	Used Sensors
1	[24]	Casilari, E.	11	Squat, going downstairs, going upstairs, jumping, jogging, getting in and out of bed, sitting and standing up from a chair, walking normally, backward fall, forward fall, and lateral fall	Accelerometer and gyroscope
2	[25]	Garcia-Gonzalez, D.	4	Inactive, active, walking (walking, running, and jogging), and driving	Accelerometer, magnetometer, GPS, and gyroscope
3	[26]	Zhang, M.	12	Walking forward, turning left, turning right, going upstairs, going downstairs, running forward, jumping, sitting, standing, sleeping, going up in an elevator, and going down in an elevator	Accelerometer and gyroscope
4	[27]	Department of Computer & Information Science	6	Walking, running, going upstairs, going downstairs, sitting, and standing	Accelerometer
5	[28]	Reiss, A.	19	Lying down, sitting, standing, walking, running, cycling, Nordic walking, watching TV, working on the computer, driving the car, going upstairs, going downstairs, vacuuming, ironing, folding clothes, cleaning home, playing soccer, jumping rope, other (transitional activities)	Accelerometer, magnetometer, gyroscope, temperature, and heart rate
6	[29]	Leutheuser, H.	13	Sitting, lying down, standing, washing dishes, vacuuming, sweeping, walking outdoors, climbing stairs, descending stairs, running on the treadmill (8.3 km/h), bicycle (50 watts), bicycle (100 watts), and jump rope	Accelerometer and gyroscope
7	[30]	Xmouyang	3	Walking in the hallway, walking, going down the stairs, and going up the stairs	Accelerometer, magnetometer, and gyroscope
8	[31]	Xmouyang	5	Walking, jumping, calling, waving (waving your hand), and typing on the cell phone	Accelerometer, magnetometer, and gyroscope
9	[32]	Reyes-Ortiz, J.	12	Walking, going up the stairs, going down the stairs, sitting, standing, lying down, standing to sitting, sitting to standing, sitting to lying down, lying to sitting, standing to lying down, lying to standing	Accelerometer and gyroscope
10	[33]	Reyes-Ortiz, J.	6	Walking, going downstairs, going upstairs, sitting, standing, and lying down	Accelerometer and gyroscope
11	[34]	Ruzzon, M.	9	Walk, sit, stand, open the door, close the door, pour water, drink from a glass, brush your teeth, and wipe the table	Accelerometer and gyroscope
12	[35]	Barshan, B.	19	Sitting, standing, lying on back, lying on right side, climbing stairs, going downstairs, standing in an elevator without moving, moving in the elevator, walking in a parking lot, walking on a treadmill at 4 km/h in a flat position, walking on a treadmill at 4 km/h with an incline of 15 degrees, running on a treadmill at 8 km/h, exercising on a stepper, exercising on an elliptical, riding a stationary bike in a horizontal position, riding a stationary bike upright, rowing, jumping, and playing basketball	Accelerometer, magnetometer, and gyroscope
13	[36]	Weiss, G.	18	Walking, jogging, stairs, sitting, standing, typing on the cell phone, brushing teeth, eating soup, eating packet potatoes, eating pasta, drinking a drink, eating a sandwich, kicking a ball, playing catch with a tennis ball, dribbling (basketball), writing, clapping, and folding clothes	Accelerometer and gyroscope
14	[37]	Davis, K.	6	Standing, sitting, lying down, walking, going up and down the stairs	Accelerometer and gyroscope
15	[38]	Pires, I.	3	Driving, sleeping, and watching television	Accelerometer, magnetometer, and gyroscope
16	[39]	Vaizman, Y.	116	Lying, sitting, standing in one place, standing, moving, walking, running, and cycling. Additionally, 109 secondary activities	Accelerometer, magnetometer, GPS, gyroscope, audio, and others.
17	[40]	Ceron, J.D	7	Walking, stairs, sitting still, using a jug, sweeping, using the sink, and using the toilet	Accelerometer and gyroscope
18	[41]	Ceron, J.D	7	Enter the apartment (go down the stairs, open the door, and enter), take off your jacket (enter the room and leave the jacket there), help yourself to something to eat and go to the dining room to eat, sweep (take the broom from the bathroom, sweep the room, and return the broom), comb your hair (go to the bathroom, take the comb, and comb your hair), go to the bathroom (raise the lid and sit on the toilet), and leave the apartment (get your jacket, take it, and leave)	Accelerometer and gyroscope
19	[42]	Ceron, J.D	3	Walking, jogging, and running	Accelerometer and gyroscope
20	[43]	Saha, S.S.	10	Walking, sitting, lying down, running, climbing stairs, going downstairs, standing, falling due to unconsciousness, falling due to a heart attack, and falling due to slipping while walking	Accelerometer

**Table 6:** Most common activities per dataset.

Dataset Number	Reference	First Author	Go Downstairs	Walk	Laying Down	Stand	Sit	Climbing Stairs
1	[24]	Casilari, E.	X	X				X
2	[25]	Garcia-Gonzalez, D.		X				
3	[26]	Zhang, M.	X			X	X	X
4	[27]	Department of Computer & Information Science	X	X		X	X	X
5	[28]	Reiss, A.	X	X	X	X	X	X
6	[29]	Leutheuser, H.	X		X	X	X	X
7	[30]	Xmouyang						
8	[31]	Xmouyang		X				
9	[32]	Reyes-Ortiz, J.	X	X	X	X	X	X
10	[33]	Reyes-Ortiz, J.	X	X	X	X	X	X
11	[34]	Ruzzon, M.		X				
12	[35]	Barshan, B.	X			X	X	X
13	[36]	Weiss, G.		X		X	X	
14	[37]	Davis, K.	X	X	X	X	X	X
15	[38]	Pires, I.						
16	[39]	Vaizman, Y.		X	X		X	
17	[40]	Ceron, J.D		X				
18	[41]	Ceron, J.D						
19	[42]	Ceron, J.D		X				
20	[43]	Saha, S.S.	X	X	X	X	X	X

X means that the dataset included the respective activity.

**Table 8:** Code column composition.

Code Segment	Options	Option Value
Sensor type	Gyroscope	1
	Both	2
	Accelerometer	3
Location side	No information	0
	Right	1
	Left	2
Sensor location	No information	0
	Wrist	1
	Hip	2
	Foot	3
	Chest	4
	Back	5
	Arm	6
	Leg	7
	Waist	8
	Other	9

## Data Availability

The data presented in this study are available on request from the corresponding author.
